# Supplementary material for: Evidence-based intrapartum care practice and associated factors among obstetric care providers working in hospitals of the four Wollega Zones, Oromia, Ethiopia
Source: PLoS One. 2023 Jan 26;18(1):e0275506. doi: 10.1371/journal.pone.0275506 (PMC9879444; doi:10.1371/journal.pone.0275506)
Supplement: S1 Checklist — (DOCX) [file pone.0275506.s001.docx]

**Observational checklist for evidence based intra-partum practice**

Data collector name________________ Signature______ Date__________

Supervisor name_________________ Signature ______Date___________

Name of the hospital Checklist code______

Profession of the care provider

**Instruction:** Observe carefully the care providers while they are providing the care and encircle “**1”** if you observe the listed practices are performed and encircle “**0”** if not performed.

| **I. Care throughout labour and birth** | | | | |
| --- | --- | --- | --- | --- |
| Care option | Description | | | **Response** |
| Respectful maternity care | | **5001**. refers to care organized for and provided to all women in a manner that maintains their dignity, privacy and confidentiality, ensures freedom from harm and mistreatment, and enables informed choice and continuous support during labour and childbirth | 1. Yes  0. No | |
| Effective communication | | **5002**. Effective communication between maternity care providers and women in labour, using simple and culturally acceptable methods | 1. Yes  0. No | |
| Companionship during labour and childbirth | | **5003.** A companion of choice throughout labour and childbirth. | 1. Yes  0. No | |

| \| **II. First stage of labour** \|  \| \| --- \| --- \| | | | |  |
| --- | --- | --- | --- | --- | --- | --- |
| Care option | Description | | Response |  |
| Clinical pelvimetry on admission | | **5004.** Routine clinical pelvimetry on admission in labour for healthy pregnant women. | 1. Yes  0. No | |
| Perineal/pubic shaving | | **5005**. Routine perineal/pubic shaving prior to giving vaginal birth | 1. Yes  0. No | |
| Digital vaginal examination | | **5006**. Digital vaginal examination at intervals of four hours for routine assessment of active first stage of labour in low-risk women. | 1. Yes  0. No | |
| Continuous cardiotocography during labour | | **5007**. Continuous cardiotocography for assessment of fetal well-being in healthy pregnant women undergoing spontaneous labour. | 1. Yes  0. No | |
| Intermittent fetal heart rate  auscultation during  labour | | **5008**. Intermittent auscultation of the fetal heart rate with either a Doppler ultrasound device or Pinard fetal stethoscope for healthy pregnant women in labour. | 1. Yes  0. No | |
| Epidural analgesia for pain relief | | **5009.** Epidural analgesia for healthy pregnant women requesting pain relief during labour depending on a woman’s preferences. | 1. Yes  0. No | |
| Opioid analgesia for pain relief | | **5010**. Parenteral opioids, such as fentanyl, diamorphine and pethidine for healthy pregnant women requesting pain relief during labour, depending on a woman’s preferences. | 1. Yes  0. No | |
| Relaxation techniques for pain management | | **5011**. Relaxation techniques, including progressive muscle relaxation, breathing, music, mindfulness and other techniques, for healthy pregnant women requesting pain relief during labour, depending on a woman’s preferences. | 1. Yes  0. No | |
| Manual techniques for pain management | | **5012**. Manual techniques, such as massage or application of warm packs for healthy pregnant women requesting pain relief during labour, depending on a woman’s preferences. | 1. Yes  0. No | |
| Oral fluid and food | | **5013**. For women at low risk, oral fluid and food intake during labour . | 1. Yes  0. No | |
| Maternal mobility and position | | **5014**. Encouraging the adoption of mobility and an upright position during labour in women at low risk . | 1. Yes  0. No | |
| Vaginal cleansing | | **5015**. Routine vaginal cleansing during labour for the purpose of preventing infectious morbidities . | 1. Yes  0. No | |

| Routine amniotomy | **5016**. The use of amniotomy alone for prevention of delay in labour . | 1. Yes  0. No |
| --- | --- | --- |
| Oxytocin for women with epidural analgesia | **5017.** The use of oxytocin for prevention of delay in labour in women receiving epidural analgesia. | 1. Yes  0. No |
| Antispasmodic agents | **5018**. The use of antispasmodic agents for prevention of delay in labour . | 1. Yes  0. No |
| Intravenous fluids for preventing labour delay | **5019**. The use of intravenous fluids with the aim of shortening the duration of labour . | 1. Yes  0. No |
| Birth position | **5020**. Encouraging the adoption of a birth position of the individual woman’s choice, including upright positions. | 1. Yes  0. No |
| Method of pushing | **5021**. Women in the expulsive phase of the second stage of labour should be encouraged and supported to follow their own urge to push. | 1. Yes  0. No |
| Techniques for preventing perineal trauma | **5022.** For women in the second stage of labour, techniques to reduce perineal trauma and facilitate spontaneous birth (including perineal massage, warm compresses and a “hands on” guarding of the perineum), based on a woman’s preferences and available options. | 1. Yes  0. No |
| Episiotomy | **5023**. Routine or liberal use of episiotomy for women undergoing spontaneous vaginal birth. | 1. Yes  0. No |
| Fundal pressure | **5024.** Application of manual fundal pressure to facilitate childbirth during the second stage of labour . | 1. Yes  0. No |

| **IV. Third stage of labour** | | | |
| --- | --- | --- | --- |
| Care option | **Description** | | Response |
| Prophylactic uterotonics | | **5025.** The use of uterotonics for the prevention of postpartum haemorrhage (PPH) during the third stage of labour. | 1. Yes  0. No |
| Delayed umbilical cord clamping | | **5026.** Delayed umbilical cord clamping (not earlier than 1 minute after birth). | 1. Yes  0. No |
| Controlled cord traction (CCT) | | **5027.** In settings where skilled birth attendants are available, controlled cord traction (CCT) is recommended for vaginal births if the care provider and the parturient woman regard a small reduction in blood loss and a small reduction in the duration of the third stage of labour as important. | 1. Yes  0. No |
| Uterine massage | | **5028.** Sustained uterine massage as an intervention to prevent postpartum haemorrhage (PPH) in women who have received prophylactic oxytocin. | 1. Yes  0. No |
| Routine nasal or oral suction | | **5029.** In neonates born through clear amniotic fluid who start breathing on their own after birth. | 1. Yes  0. No |
| Skin-to-skin contact | | **5030**. Newborns without complications kept in skin-to-skin contact (SSC) with their mothers during the first hour after birth. | 1. Yes  0. No |
| Breastfeeding | | **5031.** All newborns, including low-birth-weight (LBW) babies who are able to breastfeed put to the breast as soon as possible after birth when they are clinically stable, and the mother and baby are ready. | 1. Yes  0. No |
| prophylaxis vitamin K | | **5032.** 1 mg of vitamin K intramuscularly after birth (i.e. after the first hour by which the infant should be in skin-to-skin contact with the mother and breastfeeding should be initiated). | 1. Yes  0. No |

|  | **VI. Care of the woman after birth** | | |
| --- | --- | --- | --- |
| Care option | | Description | Response |
| Uterine tonus assessment | | **5033.** Postpartum abdominal uterine tonus assessment for early identification of uterine atony is recommended for all women. | 1. Yes  0. No |
| Antibiotics for uncomplicated  vaginal birth | | **5034.** Routine antibiotic prophylaxis for women with uncomplicated vaginal birth. | 1. Yes  0. No |
| Routine antibiotic prophylaxis for episiotomy | | **5035**. Routine antibiotic prophylaxis for women with episiotomy. | 1. Yes  0. No |
| Routine postpartum maternal assessment | | **5036**. Regular assessment of vaginal bleeding, uterine contraction, fundal height, Blood pressure , temperature and heart rate (pulse) routinely during the first 24 hours starting from the first hour after birth. | 1. Yes  0. No |
