# Supplementary material for: Evidence-based intrapartum care practice and associated factors among obstetric care providers working in hospitals of the four Wollega Zones, Oromia, Ethiopia
Source: PLoS One. 2023 Jan 26;18(1):e0275506. doi: 10.1371/journal.pone.0275506 (PMC9879444; doi:10.1371/journal.pone.0275506)
Supplement: S1 File — (DOCX) [file pone.0275506.s002.docx]

**Supporting information**

**S1 Fig.** Schematic representation of sampling Procedure in hospitals of Wollega Zones, Oromia, Ethiopia, January 20 to April 20/2022

**S2 Fig**. Magnitude of Evidence-based intrapartum care practice among obstetric care providers working in hospitals of Wollega zones, Ethiopia, January 20 to April 20, 2022 (n = 278).

**S3 Fig.** Magnitude of recommended intrapartum care practices among obstetric care providers working in hospitals of Wollega zones, Ethiopia, January 20 to April 20, 2022 (n = 278).

**S4 Fig.** Magnitude of non-recommended intrapartum care practices in hospitals of Wollega zones, Ethiopia, January 20 to April 20, 2022 (n = 278).

**S5 Fig.** Obstetric care provider’s understanding of evidence-based intrapartum care in hospitals of wollega zones, Ethiopia, January 20 to April 20, 2022 (n = 278)

**S6 Fig.** Obstetric care provider’s source of health information in Hospitals of wollega zones, Ethiopia, 2022 (n = 278)

**S7 Fig.** Percentage of average number of obstetric care providers and deliveries in Hospitals of wollega zones, Ethiopia, January 20 to April 20, 2022 (n=278)

**S1 Table**. Summary of sample size determination using double population proportion formula to study evidence based intrapartum care practice and associated factors among obstetric care providers working in hospitals of Wollega zones, Oromia, Ethiopia, 2022

**S2 Table**. Socio-demographic characteristics of obstetric care providers working in hospitals of Wollega zones, Ethiopia, January 20 to April 20, 2022 (n = 278).

**S3 Table.** Obstetric care providers level of knowledge and attitude based on their profession in hospitals of wollega zones, Ethiopia, January 20 to April 20, 2022 (n = 278)

**S4 Table**. Factors associated with Evidence-based intrapartum Care Practice in bivariable logistic regression among obstetric care providers working in hospitals of wollega zones, Ethiopia, January 20 to April 20, 2022 (n=278)

**S5 Table**. Significantly associated variables with evidence-based intrapartum care practice in a multivariable logistic regression among obstetric care providers in hospitals of wollega zones, Ethiopia, January 20 to April 20, 2022 (n=278)
