# Supplementary material for: Evidence-based intrapartum care practice and associated factors among obstetric care providers working in hospitals of the four Wollega Zones, Oromia, Ethiopia
Source: PLoS One. 2023 Jan 26;18(1):e0275506. doi: 10.1371/journal.pone.0275506 (PMC9879444; doi:10.1371/journal.pone.0275506)
Supplement: Attachment — Submitted filename: Response to Reviewers.docx [file pone.0275506.s004.docx]

# Response to Reviewers

**Dear Editors and Reviewers,**

The authors would like to forward our deepest gratitude and appreciation for devoting your valuable time to review our manuscript entitled "Evidence-based intrapartum care practice and associated factors among obstetric care providers working in hospitals of the four Wollega Zones, Oromia, Ethiopia”.

Even though evidence-based practice (EBP) is a key component of quality of intrapartum care and links to improved health care outcomes, consistent application of EBP in patient care remains a challenge for health care providers. Recent studies highlight limited application of generally accepted evidence-based clinical practices as well as limited application of more recent discoveries and recommendations. Globally, intrapartum care related complications are the leading cause of maternal and neonatal death often as a consequence of poor implementation of evidence based care practice. According to 2017 WHO estimation, everyday approximately 810 women died from causes related to poor implementation of intrapartum care practice in which Sub-Saharan Africa accounts for more than two-thirds of all these maternal deaths. In Ethiopia, maternal mortality rate (MMR) of 412 per 100,000 live births and a slight increase in neonatal mortality since 2016 from 29 to 33 deaths per 1,000 live births is still too high with possible causes from intrapartum related complications such as hemorrhage, obstructed labour or sepsis. Therefore, this study was aimed to assess magnitude of evidence-based Intrapartum care practice and associated factors among obstetric care providers working in hospitals of the four Wollega Zones, Oromia, Ethiopia.

Dear Editor, we tried to resubmit a revised minor revision of the manuscript that addresses the points raised during the review process. We include the following items in the revised manuscript as per your comments:

- A rebuttal letter that responds to each point raised by the academic editor and reviewer(s). We uploaded this letter as a separate file labeled 'Response to Reviewers'.
- A marked-up copy of the manuscript that highlights changes made to the original version. We uploaded this as a separate file labeled 'Revised Manuscript with Track Changes' colored in green.
- An unmarked version of revised paper without tracked changes. We uploaded this as a separate file labeled 'Manuscript'.
- Regarding financial disclosure, we included updated statement in the cover letter.

We have also addressed these additional journal requirements.

1. We ensured that the manuscript meets PLOS ONE's style requirements, including those for file naming.
2. The grant information provided in the funding information and financial disclosure section is now matched.
3. The authors received no specific funding for this work. The amended statements are included within the manuscript; you can change the online submission form.
4. Thank you for the remark. There are no restrictions and the anonymized data set is uploaded as a supporting information file. We would like to mention that anybody who wants the data set can get it from the corresponding author through an email address; otherwise there are no ethical and legal restrictions. Now as PLOS standard we had uploaded the minimal anonymized data set. You can update the data availability statement.
5. Concerning repository information we provided for data at acceptance; if the manuscript has accepted for publication, please don’t hold it until we provide the relevant accession numbers or DOIs necessary to access the data. We wish to make changes to Data Availability statement. We described these changes in the cover letter and you can update data availability statement to reflect the information we provide.
6. Captions for supporting information files are included at the end of the manuscript.
7. References are reviewed and corrected

Again, we would like to remind our deepest gratitude for your effort for the improvement of this manuscript.

Kind regards,

Seid Wodajo on the behalf of co-authors

## Reviewer Comments to the Authors

**Reviewer #1:**

**Comment:** The manuscript was a questionnaire based survey conducted on health care workers and it was on their knowledge and practices of the World Health Organization evidence based practices of intrapartum care of women, within Healthcare facilities in Ethiopia. The work has been well written and it is a relevant aspect in Obstetric care.

**Response**: Thank you for your interest on the study and approving its relevant aspect in obstetric care.

**Comment:** The introduction was quite long and it will be good if it can be reasonably and more concisely presented.

**Response:** accepted and revision was made. We removed one paragraph reasonably.

**Comment:** The Authors should take note of typographical and text edit errors and advice on use of plagiarism checker for example, "While some of the intrapartum care classified as not recommended practices includes routine assessment of fetal well-being on labor admission, Perineal/pubic shaving, Continuous cardiotocography during labor, Routine amniotomy and Intravenous fluids for preventing labor delay (8)." (page 10, line 1).

**Response:** accepted and revision was made. Now it is rewritten as “On the other hand, Some of the intrapartum care practices classified as not recommended practices including routine assessment of fetal well-being on labor admission, Perineal/pubic shaving, Continuous cardiotocography during labor, Routine amniotomy and Intravenous fluids for preventing labor delay (6)”.

**Comment**: The referencing method was haphazardly done and uniform pattern will be appreciated.

**Response:** accepted and revision was made.

**Comment:** The P-value of <0.05, had been stated as being statistically for the determination of statistical significance for their data but it was noted that values above this (0.25), had been used for inclusion of variables in the multivariate analysis. This should be reviewed by the Authors to reduce bias. The service of a statistician can be helpful.

**Response:** It has been reviewed by the Authors with the help of statisticians. Thank you for your scholarly comments and suggestions.

**Comment**: In the results section both texts and tables should not be used to present the results to reduce duplication of information.

**Response:** Not all the information from the table was presented in the form of text. Only some of the main findings were taken and explained to give emphasize and to make it more understandable.

**Comment:** The conclusion was also too long and should be abridged.

**Response:** accepted and revision was made.

**Comment:** The references should also be updated, as over 10% of the references appeared old.

**Response:** Some of the references are updated.

**Remark:** The manuscript was well done and if these comments are noted and revised, the work is publishable.

**Response:** The comments are well noted and revised as much as we can. We really thank you for your scholarly comments and suggestions as well as for giving us .

Kind Regards,

Seid Wodajo on the behalf of co-authors

**Reviewer #2:** The manuscript is well written is simple English language that was easy to understand. The topic is of contemporary relevance that will add to the body of knowledge. However, it requires minor revision in view of the points highlighted below:

**Response:** All the authors thanked you and are pleased with your scholarly comments and suggestions as well as for approving the relevance of the topic to the body of knowledge.

**Comment:** The running title is too long and may be shortened as suggested in the manuscript

**Response:** accepted and revised as “Evidence-based intrapartum care practice and associated factors among obstetric care providers working in hospitals of the four Wollega Zones, Oromia, Ethiopia”.

**Comment:** The detail of statistical analysis need not appear in the abstract

**Response:** accepted and revision was made.

**Comment:** On page 17, the third sentence in results under socio-demographic characteristics needs modification thus: ‘I suggest that this should be reported in the context of positive Evidence-based intrapartum care practice and not overall respondent’.

**Response:** It is modified as “There was a male predominance 119 (63.7%) and 162 (58.3%) of the care providers were married”. This figure (109 (67.3%) was the number/percentage of married respondents who were practicing evidence-based intrapartum care not the overall married respondents. The overall frequency/percentage of married respondents was revised as 162 (58.3%) as stated above.

**Comment:** On page 21 under ‘Factors associated with Evidence-based intrapartum care practice in a bivariable logistic regression’: The sentence is too long. Please break them into two for ease of understanding.

**Response:** accepted and revised.

**Comment:** On page 25, the second paragraph the Authors should try and harmonize the possible reasons behind their lower findings compared to earlier studies.

**Response:** accepted and revised.

**Comment:** On page 26, second paragraph, the highlighted sentence should be replaced with ‘was in contrast with the findings of Walsh D in London (32)’.

**Response:** accepted and replaced.

**Comment:** On page 27, under limitation: There was no mention of the smaller study population compared to the actual calculated sample size as a possible limitation.

**Response:** Well accepted and revised**.** Thank you for showing us this significant limitation of the study. This was the main reason for using census method due to the smaller study population compared to the actual calculated sample size as mentioned in methodology section within the manuscript.

**Comment:** Concerning the second sentence on page 28, ‘While routine amniotomy was not a recommended practice according to WHO guidelines, is it really a harmful practice?’

**Response:** Yes, obviously. Routine amniotomy is one of the non-recommended/harmful practices according to the latest WHO recommendations on intrapartum care for a positive childbirth experience (2018).

**Comment:** On page 29, references 3 and 4 were repetitions.

**Response:** Corrected

**Comment:** On same page 29, reference 5 and 6 should be properly cited

**Response:** accepted and corrected

- PLOS authors have the option to publish the peer review history of their. If published, this will include your full peer review and any attached files.
  Do you want your identity to be public for this peer review**?**

**Response: #YES**

Dear reviewers, we hope now the manuscript is clear and more acceptable than the previous one. We have tried to present the manuscript in proper manner according to your comments. Finally, we would like to extend our deepest gratitude for your endeavor for the improvement of this manuscript.

Kind regards,

Seid Wodajo on the behalf of co-authors
